# Supplementary material for: Effect of smoking on drug-resistant tuberculosis treatment outcomes and potential mechanistic pathways: a multicountry cohort study
Source: BMJ Open Respir Res. 2025 Dec 25;12(1):e003777. doi: 10.1136/bmjresp-2025-003777 (PMC12742115; doi:10.1136/bmjresp-2025-003777)
Supplement: online supplemental file 1 [file bmjresp-12-1-s001.docx]

**SUPPLEMENTARY TABLES**

**Supplementary Table 1. Rationale for each exclusion criterion for the analytic study population**

| **Exclusion criterion** | **Rationale** |
| --- | --- |
| Treated in the Democratic People’s Republic of Korea | Compared with other sites, the Democratic People’s Republic of Korea had major differences in clinical protocols (e.g., no HIV testing) and regimens, including shortened treatments. For these reasons, other analyses of endTB Observational Study data have typically excluded this country. |
| Had confirmed rifampicin-susceptible TB | Our analysis was focused on people with MDR/RR-TB. Some participants, particularly when culture-based methods for resistance were used, may have experienced a delay between cohort enrollment and obtaining rifampicin resistance results. |
| Had only extrapulmonary TB | The biologic pathways in which smoking affects TB treatment outcomes for extrapulmonary TB may differ from those for pulmonary TB (lung damage and impaired pulmonary immune response). Some variables used in our analyses are not applicable to people without pulmonary TB (i.e., sputum smear and culture positivity, presence of cavitary disease). |
| Enrolled in the endTB Observational Study more than one month after MDR/RR-TB treatment initiation | Some participants in the endTB Observational Study were enrolled later in the course of MDR/RR-TB treatment and their time of observation would not align with that of the majority of participants who started treatment around the time of cohort enrollment. This was an important consideration because of how the exposure was ascertained (smoking at baseline, i.e., start of MDR/RR-TB treatment vs. later during treatment) and our longitudinal analyses, which included time-varying variables. |
| Were under 15 years of age | Adults and older adolescents (≥15 years of age) comprise the vast majority of people who smoke cigarettes daily and, therefore, are our target population. Furthermore, reasons for lost to follow-up for younger participants (0–14 years of age) are expected to be different from those for older participants and likely involve their parents or caregivers. |
| Treated at a site with no participants who reported smoking | Recognizing that there are both measured and unmeasured differences in participant characteristics among sites, we sought to include sites that had at least some variation in the exposure (smoking). |
| Treated at a site that did not collect adherence data or had inconsistent reporting | Since we expected TB treatment adherence to be strongly predictive of loss to follow-up, it was used in our computation of inverse probability of censoring weights. We would not be able to compute these weights for participants at sites that did not collect adherence data, and we would not be able to accurately compute these weights for participants at sites with inconsistent reporting. |
| Transferred out or had an unknown end-of-treatment outcome | This small number of participants would be censored in our analyses since their MDR/RR-TB outcome was unknown. Their exclusion from the analytic sample is unlikely to influence our results. |

**Supplementary Table 2. Selected characteristics of endTB Observational Study participants stratified by inclusion and exclusion into our analytic study population**

| **Characteristic at cohort enrollment** | **n** | **All included,***  n=1786 | **n** | **Excluded, but at same sites as included, ^†^** n=260 |
| --- | --- | --- | --- | --- |
|  |  | n (%) |  | n (%) |
| Post-Soviet country | 1786 | 1056 (59.1) | 260 | 141 (54.2) |
| Male sex | 1786 | 1136 (63.6) | 260 | 159 (61.2) |
| Married or living with partner | 1780 | 919 (51.6) | 255 | 111 (43.5) |
| Employed | 1777 | 270 (15.2) | 251 | 39 (15.5) |
| Homeless in past year | 1727 | 69 (4.0) | 241 | 8 (3.3) |
| Ever incarcerated | 1485 | 273 (18.4) | 203 | 35 (17.2) |
| Refugee, displaced person, or migrant | 1757 | 57 (3.2) | 239 | 9 (3.8) |
| Drinks alcohol | 1762 | 216 (12.3) | 234 | 25 (10.7) |
| Used non-prescribed illicit drugs and/or intravenous drugs in past year | 1718 | 93 (5.4) | 221 | 15 (6.8) |
| HIV | 1785 | 118 (6.6) | 260 | 31 (11.9) |
| Hepatitis B virus infection | 1782 | 73 (4.1) | 258 | 16 (6.2) |
| Hepatitis C virus infection | 1782 | 246 (13.8) | 260 | 29 (11.2) |
| Diabetes | 1785 | 243 (13.6) | 259 | 30 (11.6) |
| Bilateral pulmonary disease | 1710 | 1137 (66.5) | 241 | 138 (57.3) |
| Lung fibrosis | 1646 | 1105 (67.1) | 224 | 130 (58.0) |

*In **Table 1**. **^†^**A total of 847 participants were excluded, including all participants at four sites: Bangladesh (n=279), Haiti (n=37), Kenya (n=7), Lesotho (n=264). Because of expected differences by sites, these 587 participants were not included for this comparison.

**Supplementary Table 3. Specification of stabilized time-varying inverse probability of censoring weights**

| **Specification** | **Mean (SD)** | **Min, max** |
| --- | --- | --- |
| **Numerator:** Time;* quadratic time; smoking status (A_0_); and baseline demographics, social history, and comorbidities (L_1,0_).^†^  **Denominator:** Time;* quadratic time; smoking status (A_0_); and baseline demographics, social history, and comorbidities (L_1,0_);^†^ baseline indicators of TB disease severity (L_2,0_),^†^ time-varying measures of TB diseases severity (smear positivity, culture positivity, and cavitary disease) and TB treatment adherence (L_t_).^‡^ | 1.00 (0.11) | 0.58, 2.34 |

SD, standard deviation.

*Time defined as week of treatment.
^†^As with the regression analyses, a missing category was included in the variable and separate variable to indicate missingness was added.
^‡^For time-varying variables, observations were carried forward in time until measured again or the end of treatment. For any missing data, a missing category was included in the variable and separate variable to indicate missingness was added.
